# Probing the glioma microvasculature: a case series of the comparison between perfusion MRI and intraoperative high-frame-rate ultrafast Doppler ultrasound

**DOI:** 10.1186/s41747-023-00406-0

**Published:** 2024-01-26

**Authors:** Ahmad Alafandi, Sadaf Soloukey Tbalvandany, Fatemeh Arzanforoosh, Sebastian R. van Der Voort, Fatih Incekara, Luuk Verhoef, Esther A. H. Warnert, Pieter Kruizinga, Marion Smits

**Affiliations:** 1 Department of Radiology & Nuclear Medicine, Erasmus MC, Dr.Molewaterplein 40, 3015 GD Rotterdam, The Netherlands; 2 Brain Tumour Centre, Erasmus MC Cancer Institute, Rotterdam, The Netherlands; 3 Department of Neurosurgery, Erasmus MC, Rotterdam, The Netherlands; 4 Department of Neuroscience, Erasmus MC, Rotterdam, The Netherlands; 5 Medical Delta, Delft, The Netherlands

**Keywords:** Cerebral blood volume, Glioma, Magnetic resonance imaging, Perfusion, Ultrasonography (Doppler)

## Abstract

**Background:**

We aimed to describe the microvascular features of three types of adult-type diffuse glioma by comparing dynamic susceptibility contrast (DSC) perfusion magnetic resonance imaging (MRI) with intraoperative high-frame-rate ultrafast Doppler ultrasound.

**Methods:**

Case series of seven patients with primary brain tumours underwent both DSC perfusion MRI and intra-operative high-frame-rate ultrafast Doppler ultrasound. From the ultrasound images, three-dimensional vessel segmentation was obtained of the tumour vascular bed. Relative cerebral blood volume (rCBV) maps were generated with leakage correction and normalised to the contralateral normal-appearing white matter. From tumour histograms, median, mean, and maximum rCBV ratios were extracted.

**Results:**

Low-grade gliomas (LGGs) showed lower perfusion than high-grade gliomas (HGGs), as expected. Within the LGG subgroup, oligodendroglioma showed higher perfusion than astrocytoma. In HGG, the median rCBV ratio for glioblastoma was 3.1 while astrocytoma grade 4 showed low perfusion with a median rCBV of 1.2. On the high-frame-rate ultrafast Doppler ultrasound images, all tumours showed a range of rich and organised vascular networks with visually apparent abnormal vessels, even in LGG.

**Conclusions:**

This unique case series revealed *in vivo* insights about the microvascular architecture in both LGGs and HGGs. Ultrafast Doppler ultrasound revealed rich vascularisation, also in tumours with low perfusion at DSC MRI. These findings warrant further investigations using advanced MRI postprocessing, in particular for characterising adult-type diffuse glioma.

**Relevance statement:**

Our findings challenge the current assumption behind the estimation of relative cerebral blood volume that the distribution of blood vessels in a voxel is random.

**Key points:**

• Ultrafast Doppler ultrasound revealed rich vascularity irrespective of perfusion dynamic susceptibility contrast MRI state.

• Rich and organised vascularisation was also observed even in low-grade glioma.

• These findings challenge the assumptions for cerebral blood volume estimation with MRI.

**Graphical Abstract:**

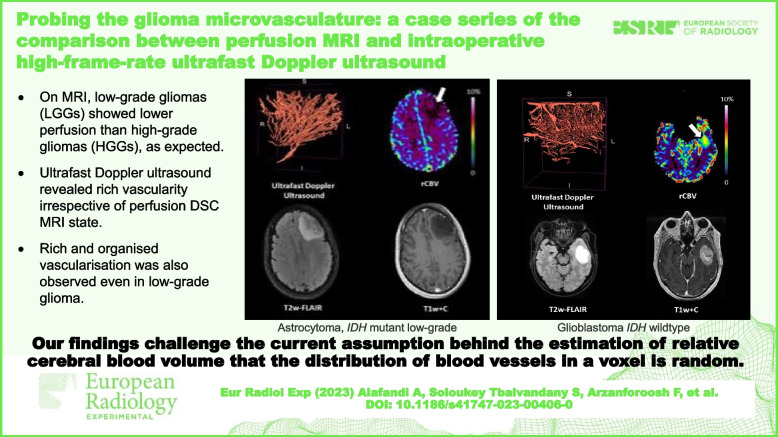

## Background

Adult-type diffuse glioma is a highly infiltrative central nervous system tumour with a prognosis that varies widely depending on the morphological and molecular features of the tumour. Due to the crucial role of molecular characterisation in glioma grading and prognostics, the recent edition of the 2021 WHO classification [1] distinguishes three types of adult-type diffuse glioma according to their genetic alterations: astrocytoma isocitrate dehydrogenase (*IDH*) mutant, oligodendroglioma *IDH* mutant and *1p/19q* codeleted, and glioblastoma *IDH*-wildtype.

Dynamic susceptibility contrast (DSC) perfusion magnetic resonance imaging (MRI) has been broadly utilised to differentiate between different grades and types of glioma using cerebral haemodynamic parameters such as cerebral blood volume (CBV) and cerebral blood flow [2–5]. In DSC MRI, the passage of the paramagnetic contrast agent induces a signal loss in the magnetic susceptibility and leads to shortening of the effective transverse relaxation time T2*. The susceptibility-induced signal loss is assumed to be proportional to the amount of contrast in the blood vessels which are assumed to be randomly oriented within the voxel. This forms the basis to generate the CBV parameter by assessing the signal intensity-time changes in the blood vessels using the kinetic tracer modelling and indicator dilution theory. Due to the semiquantitative nature of this estimation, the term “relative” CBV (rCBV) is generally used [6].

Several studies have investigated the role of DSC MRI in grading and typing brain tumours, considering that higher grade and more aggressive tumours are associated with two types of vasculature: angiogenesis and vascular proliferation [7–12]. High-grade tumours are characterised with higher perfusion (higher rCBV) whereas low-grade gliomas are characterised by low rCBV values due to the fact that the tumour vascular features are similar to the normal brain tissue without neoangiogenesis [13].

A recently published study assessed the clinical potential of functional ultrasound (fUS) during awake craniotomy in vascular and functional brain mapping, including ten patients with frontal and temporal lobe tumours who underwent MRI preoperatively according to local conventional clinical protocol [14]. The novel imaging tool functional high-frame-rate ultrafast Doppler ultrasound has a higher spatiotemporal resolution compared to other imaging modalities, which allows to detect haemodynamic changes in real time and obtain robust *in vivo* images of the tumour vascular bed.

In this case series, we describe the microvascular features of the three types of adult-type diffuse glioma as a comparison between DSC perfusion MRI and intra-operative ultrafast high-frame-rate Doppler ultrasound.

## Methods

### Patients

We included ten patients with a primary brain tumour who underwent awake brain surgery at the Department of Neurosurgery of Erasmus MC (Rotterdam, The Netherlands) and were included in the previously published fUS-study [14].

Patients’ eligibility for the fUS study has been reported earlier [14]. Additionally, the following inclusion criteria for this study were set, *i.e.*, the availability of the DSC perfusion MRI scans and of the ultrafast Doppler ultrasound images. On this basis, three patients were excluded: patients #01 and #07 for missing DSC perfusion MRI scans and patient #06 due to technical issues with acquiring the ultrafast Doppler ultrasound images. Five out of the remaining seven included patients had simultaneously been included in a published study [15] (iGENE) which focused on evaluating the clinical potential of assessing tumour vasculature in the three different types of non-enhancing adult-type glioma with MRI. Patients’ clinical information was collected on age, sex, and histopathological and molecular diagnosis of the tumour.

### Ultrafast Doppler ultrasound image acquisition and preprocessing

Images were acquired intra-operatively using an experimental research system (Vantage-256, Verasonics, Kirkland, Washington, USA) interfaced with a 5-MHz, 128-element linear array transducer (ATL Philips, L7-4300-mm pitch). Image acquisition was performed continuously with an angled plane wave ranging from 12–16 angles equally spaced between -12 and 12 degrees with a pulse repetition frequency ranging from 6 to 8 kHz depending on the imaging depth. The power Doppler images were computed from an average ensemble size of 120–140 frames providing Doppler images at 3.6–4.8Hz. The raw angled-compounded beamformed frames acquired with a frame rate ranging from 500 to 667 Hz were stored on a fast hard disc for offline processing purposes. Finally, power Doppler Images were processed using MATLAB (version R2021b) following the pipeline reported in the fUS study [14].

### MRI protocol

All patient scans were acquired using a 3-T unit (MR750, General Electric Healthcare, Milwaukee, WI, USA) prior to surgery with a 32-channel head coil. For perfusion MRI, the five patients from the iGENE study had undergone a hybrid echo-planar imaging (HEPI) sequence to simultaneously collect both T2-weighted spin-echo images and T2*-weighted gradient-echo images. For this study, only the HEPI-GRE T2*-weighted images were used for calculating rCBV. Patients #05 and #08 had undergone a conventional GRE DSC sequence to acquire T2*-weighted images. All MRI perfusion scans were performed with the administration of 7.5 mmol of a gadolinium-based contrast agent (Gadovist, Bayer Healthcare, Leverkusen, Germany). A preload bolus of equal size was given five minutes prior to the DSC/HEPI acquisition (Table 1). The MRI protocol also included high-resolution structural images as part of routine clinical imaging. These were unenhanced and contrast-enhanced three-dimensional (3D) T1-weighted, two-dimensional T2-weighted, and 3D T2-weighted flow-attenuated inversion-recovery scans.

**Table 1:** Acquisition parameters for perfusion MRI

Sequence		Repetition time (ms)	Echo time (ms)	Voxel size (mm^3^)	Slice thickness (mm)/gap interslice (mm)
Hybrid echo-planar imaging (patients #02, #03, #04, #09, #10)		1,500	GRE 18.6 SE 69	1.9 ×1.9 × 4.0	3.0/1.0
DSC GRE	Patient #05	2,000	45	2.0 × 2.0 × 5.0	5.0/0.0
	Patient #08			1.9 × 1.9 × 6.0	6.0/0.0

*DSC* Dynamic susceptibility contrast, *GRE* Gradient-echo, *MRI* Magnetic resonance imaging

### Tumour mask segmentation

For tumour segmentation, the structural scans (contrast-enhanced T1-weighted, T2-weighted, and T2-weighted flow-attenuated inversion-recovery) were linearly coregistered to the unenhanced T1-weighted images using the Elastix toolbox [16]. Then, HD-BET [17] was used to create a brain mask and extract the brain. Finally, the in-house developed algorithm of Glioseg was applied to generate 3D tumour mask using five different segmentation networks [18–21].

### Vessel segmentation

For 3D visual representation of the tumour vasculature, the ultrafast Doppler ultrasound images were first stored in Neuroimaging Informatics Technology Initiative, NIfTI [https://nifti.nimh.nih.gov/], formatted, and then reviewed using 3DSlicer software for denoising. To attenuate the background noise, a low-pass Gaussian blur filter was applied and subtracted from the original image. The images were segmented using 3DSlicer [https://www.slicer.org/] by setting a minimum manual threshold value of 30 dB, then edited using smoothing segmentation tools to remove segmented components with fewer than 500 voxels.

### Relative CBV quantification

To generate rCBV maps, the T2*-weighted scans were used as input in DICOM format to IBNeuro (version 21.12, Imaging Biometrics, Elm Grove, Wisconsin, USA) software with the leakage correction feature selected due to contrast enhancement on contrast-enhanced T1-weighted images [22]. The tumour mask was used as a region of interest to compute the voxel-wise rCBV within the entire tumour. A normal-appearing white matter (NAWM) mask from the hemisphere contralateral to the tumour was extracted for rCBV normalisation, to calculate the ratio between the tumour rCBV and the NAWM rCBV. To obtain this mask, first FAST (FMRIB's Automated Segmentation Tool [23]) was applied on the brain-extracted unenhanced T1-weighted images to generate probability maps of white matter, grey matter and cerebrospinal fluid. The white matter probability map from the hemisphere contralateral the tumour was then selected and binarised (probability > 0.9). To reduce partial volume effects, the binarised map was eroded with FSL tools (http://www.fmrib.ox.ac.uk/fsl/), with a kernel size of 3 × 3 × 3 mm^3^. For this, the brain-extracted unenhanced T1-weighted images were linearly registered to the fifth brain volume of HEPI/DSC-GRE (after preprocessing) using MC FLIRT (FMRIB’s Linear registration Tool, University of Oxford, Oxford, UK) [24, 25]. Then, the same transformation matrix was used to transfer the NAWM and tumour masks to the HEPI/DSC-GRE space and binarised with FSL tools with a threshold of 0.9.

In case MC FLIRT fails to perform registration due to the small field of view of HEPI/DSC-GRE data (*n* = 3 patients), NAWM and tumour masks were only resampled via FSLEYES to HEPI/DSC-GRE space and then binarised with a threshold of 0.9. Histograms were obtained using in-house python script from which median, mean, and maximum rCBV ratios were extracted.

## Results

**Fig. 1:**
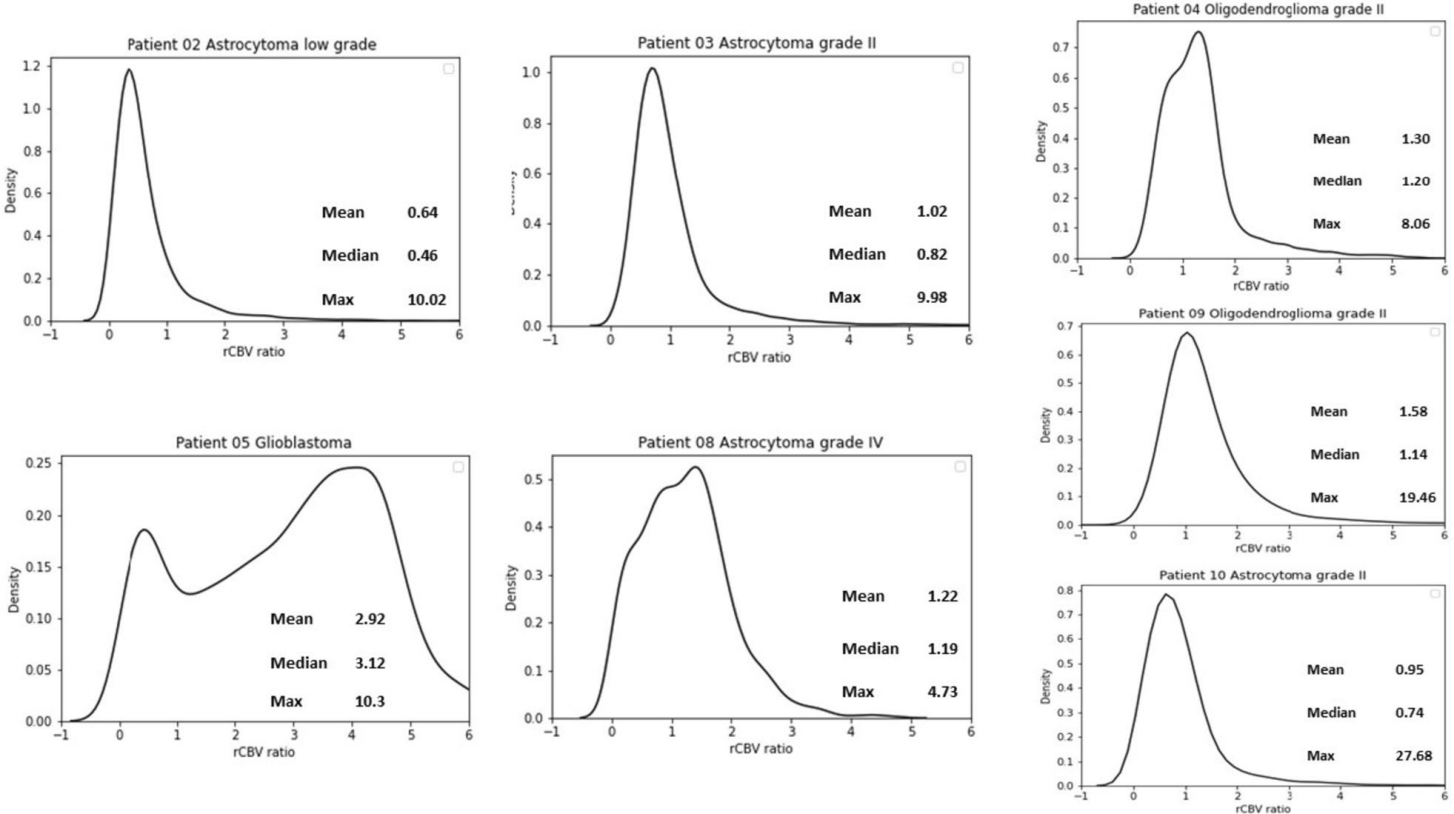
Tumour relative cerebral blood volume (rCBV) ratio histograms

From the seven included patients, two had a high-grade and five a low-grade glioma. Patient characteristics are provided in Table 2. Perfusion was high in glioblastoma and low in all low-grade tumours, as expected. The rCBV histograms, mean, median and maximum values are given in Fig. 1.

**Table 2:** Patient characteristics

Patient #	Tumour type	Molecular profile	Age (years)	Sex	Tumour volume (cm^3^)	Median rCBV ratio	Contrast enhancement	Location
02	Low-grade astrocytoma	*IDH* mutant	46	Female	61.3	0.5	No	Left frontal
03	Grade 2 astrocytoma	*IDH* mutant	40	Male	32.7	0.8	No	Left frontal
04	Grade 2 oligodendroglioma	*IDH* mutant *1p/19q* codeleted	31	Male	18.0	1.2	No	Right parietal
05	Glioblastoma	*IDH* wildtype	56	Male	137.2	3.1	Yes	Left temporal
08	Grade 4 astrocytoma	*IDH* mutant	46	Male	113.8	1.2	Yes	Right temporal
09	Grade 2 oligodendroglioma	*IDH* mutant *1p/19q* codeleted	31	Male	34.0	1.1	No	Left frontal
10	Grade 2 astrocytoma	*IDH* mutant	34	Female	67.6	0.7	No	Left frontal

*IDH* Isocitrate dehydrogenase, *rCBV* Relative cerebral blood volume

### Patient #02

**Fig. 2:**
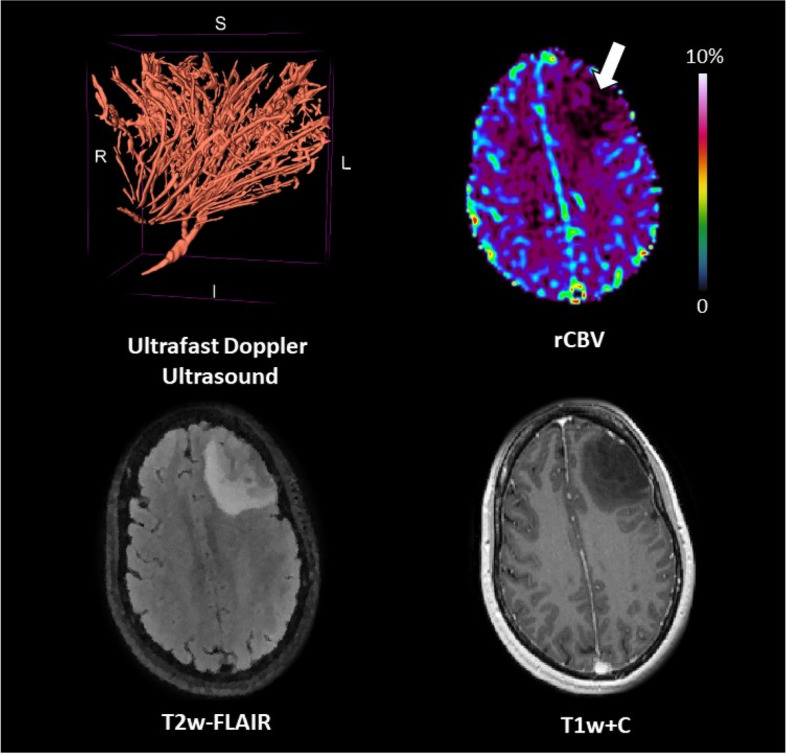
Patient #02: astrocytoma, *IDH* mutant low-grade

The patient was a 46-year-old female with low-grade non-enhancing astrocytoma *IDH*-mutant located in the left frontal lobe (tumour volume = 61.3 cm^3^). Perfusion was low (median rCBV ratio = 0.5). On ultrafast Doppler ultrasound, the vasculature of the tumour seemed to merge into one single vessel (Fig. 2).

### Patient #03

**Fig. 3:**
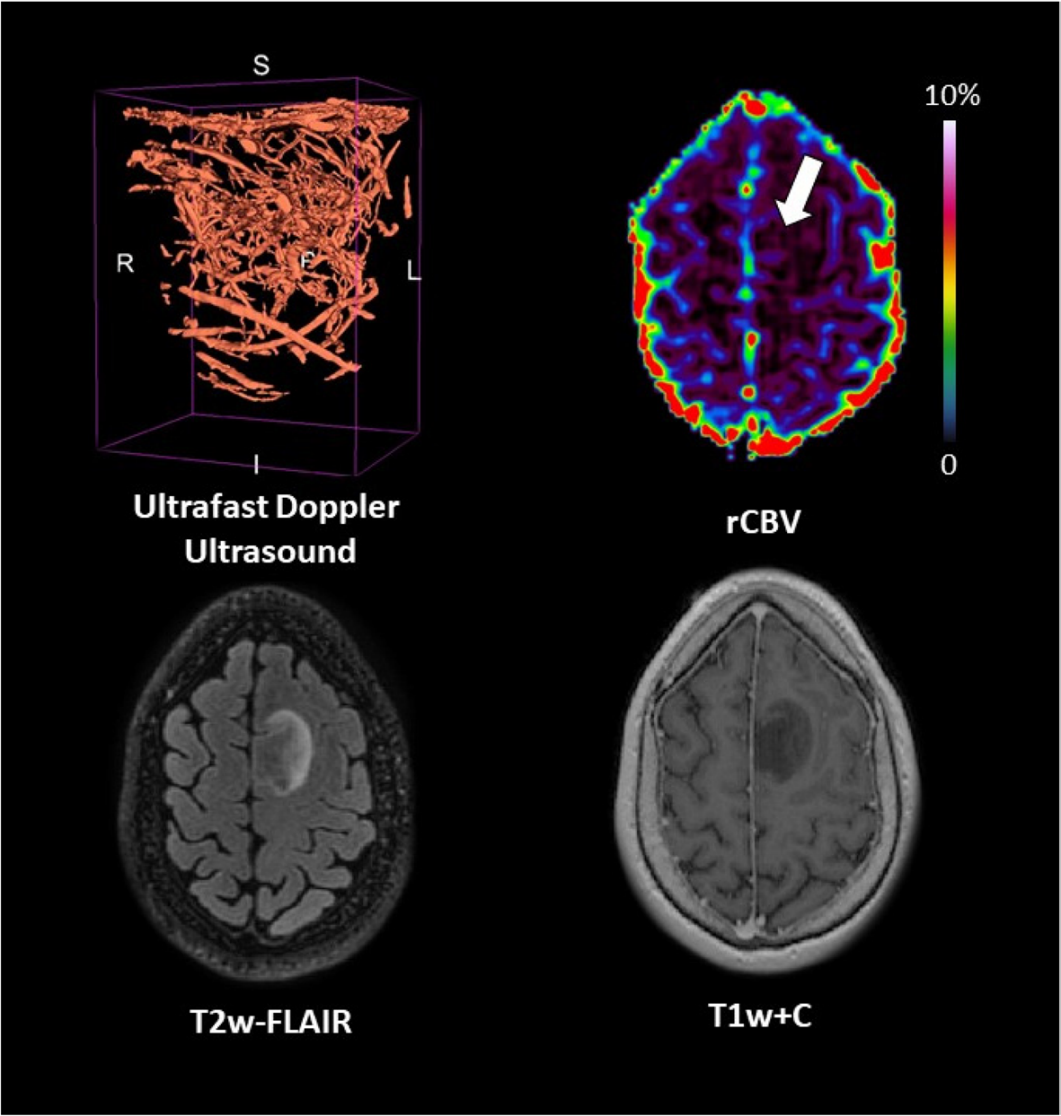
Patient #03: astrocytoma, *IDH* mutant grade 2

The patient was a 40-year-old male with a non-enhancing astrocytoma grade 2 *IDH* mutant in the left frontal lobe (tumour volume = 32.7 cm^3^). Perfusion was low (median rCBV ratio = 0.8). Ultrafast Doppler ultrasound showed a dense transversal network of tortuous vessels (Fig. 3).

### Patient #04

**Fig. 4:**
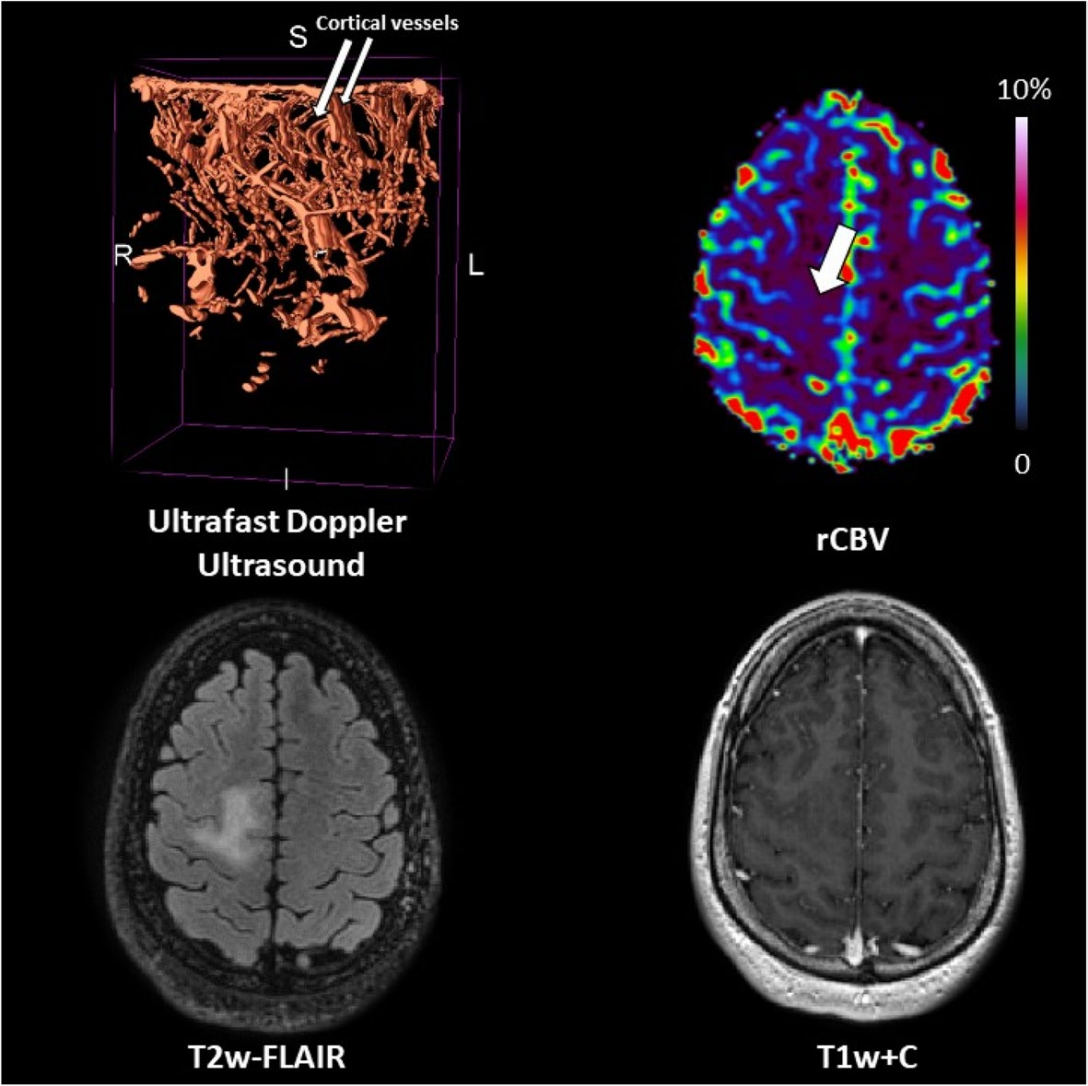
Patient #04 oligodendroglioma, *IDH* mutant *1p/19q* codeleted grade 2

The patient was a 31-year-old male with a non-enhancing grade 2 oligodendroglioma *IDH* mutant-*1p/19q* codeleted, located in the right parietal lobe (tumour volume = 18.0 cm^3^). Perfusion was low (median rCBV ratio = 1.2). Ultrafast Doppler ultrasound showed feather-like vessels with infiltrative compact vasculature as well as cortical components apparent as short and highly organised vessels penetrating the cortical anatomy (Fig. 4).

### Patient #05

**Fig. 5:**
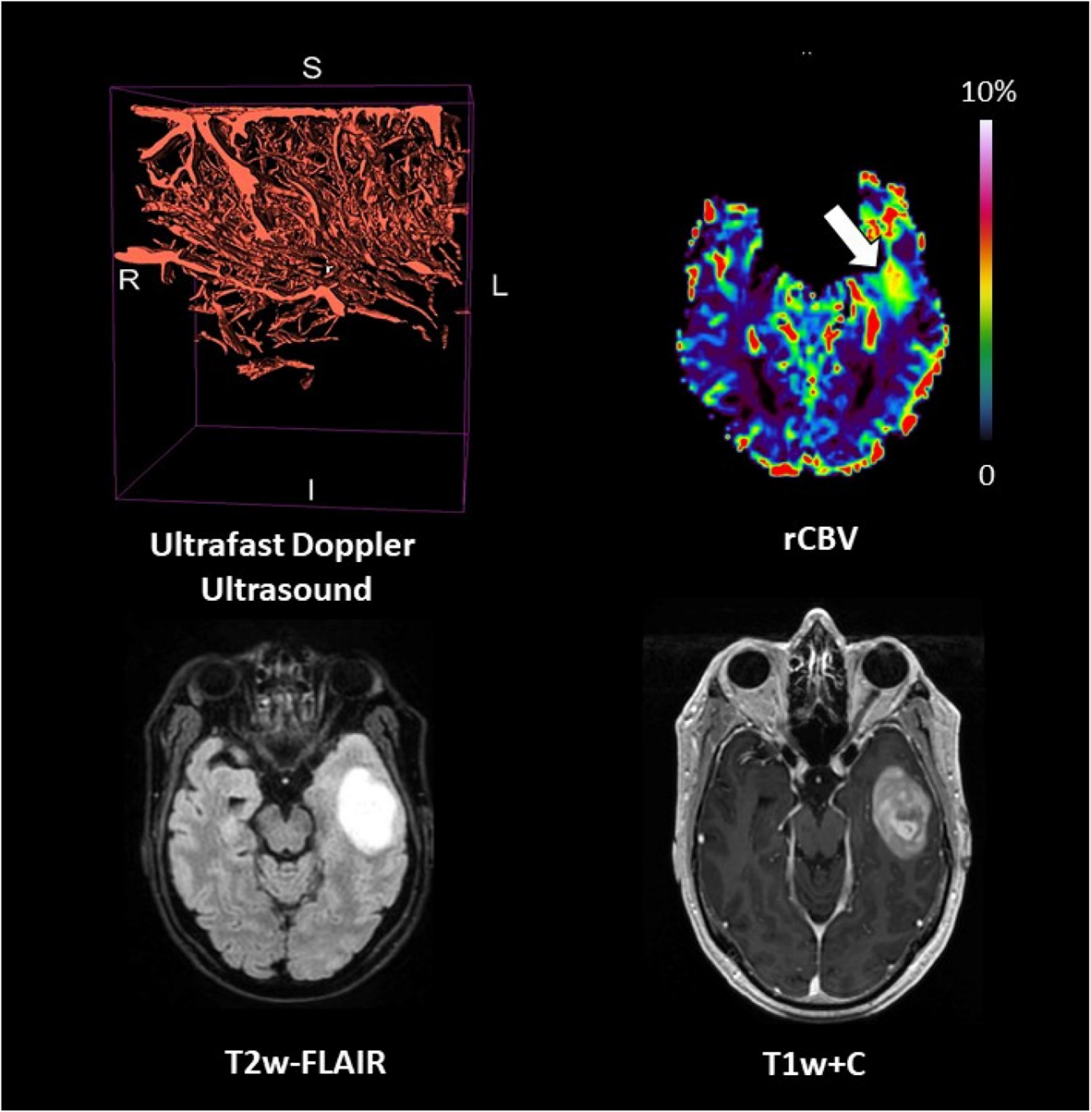
Patient #05 : glioblastoma *IDH*-wildtype

The patient was a 56-year-old male with a contrast-enhancing glioblastoma *IDH* wild-type located in the left temporal lobe (tumour volume = 137.2 cm^3^). Perfusion was high (median rCBV ratio = 3.1). Ultrafast Doppler ultrasound showed a highly dense vascular architecture with the superficial cortical vessels exposed (Fig. 5).

### Patient #08

**Fig. 6:**
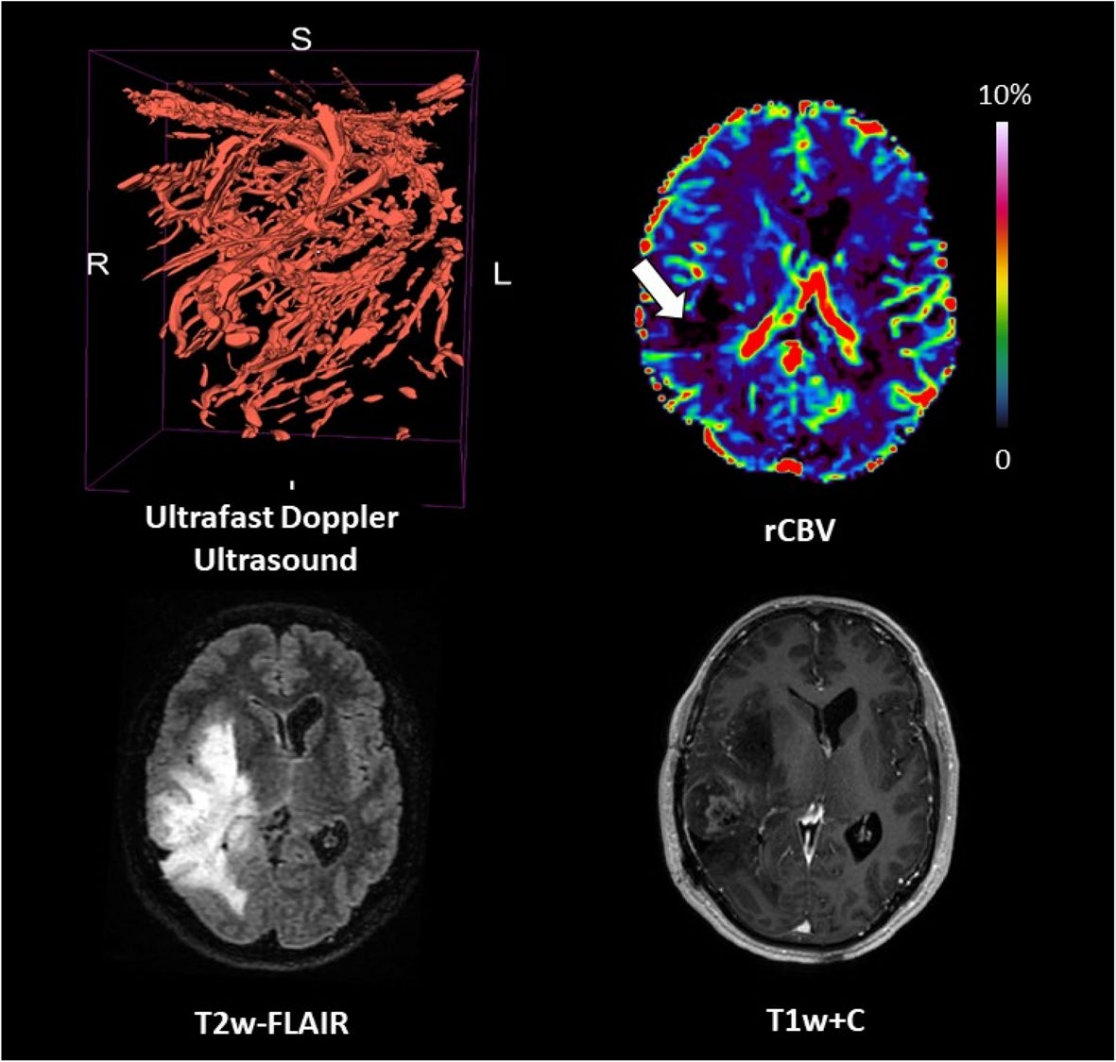
Patient #08: astrocytoma, *IDH* mutant grade 4

The patient was a 46-year-old male with an enhancing grade 4 *IDH* mutant astrocytoma, located in the right temporal lobe (tumour volume = 113.8 cm^3^). Perfusion was low (median rCBV ratio = 1.2). Ultrafast Doppler ultrasound reveals exuberant and distorted vasculature (Fig. 6).

### Patient #09

**Fig. 7:**
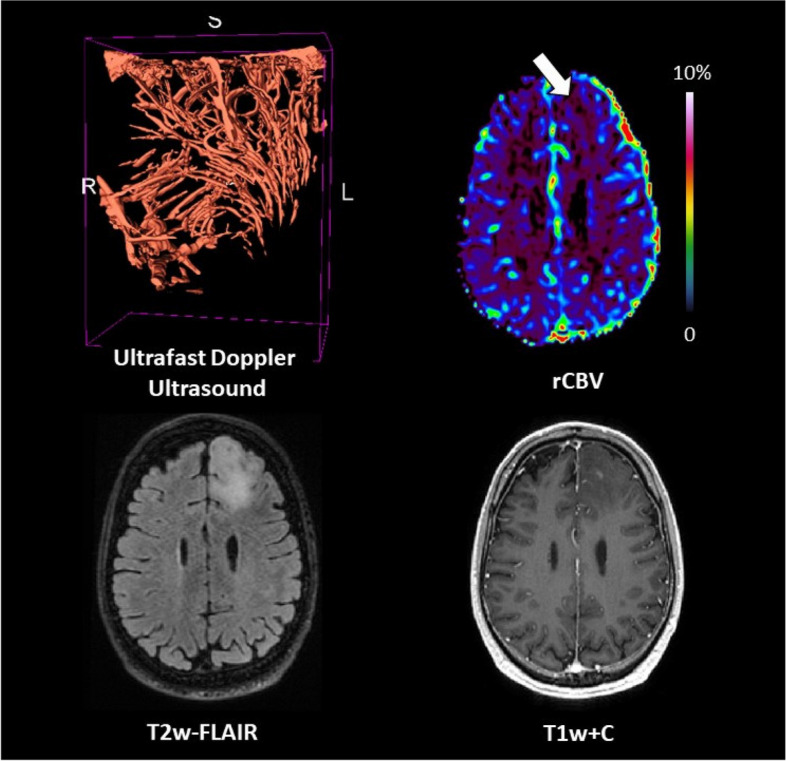
Patient #09: oligodendroglioma, *IDH* mutant *1p/19q* codeleted grade 2

The patient was a 31-year-old male with a non-enhancing oligodendroglioma grade 2 *IDH* mutant *1p/19q* codeleted, located in the left frontal lobe (tumour volume = 34.0 cm^3^). Perfusion was low (median rCBV ratio = 1.1). Ultrafast Doppler ultrasound showed long arraying vessels forming a coordinated vascular network with a circular structure (Fig. 7).

### Patient #10

**Fig. 8:**
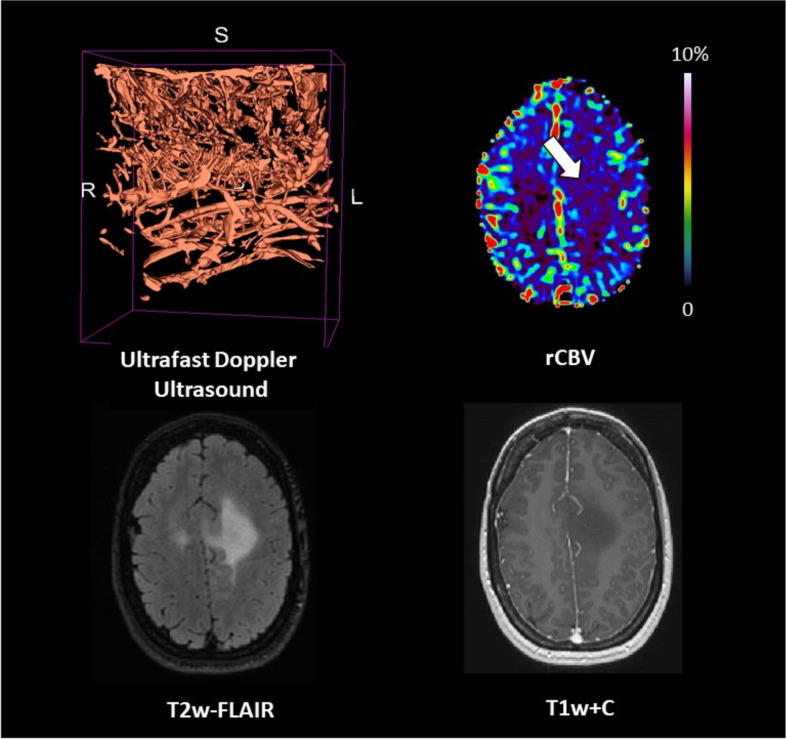
Patient #10: astrocytoma, *IDH* mutant grade 2

The patient was a 34-year-old female with a non-enhancing grade 2 *IDH* mutant astrocytoma, located in the left frontal lobe (tumour volume = 67.6 cm^3^). Perfusion was low (median rCBV ratio = 0.7). Ultrafast Doppler ultrasound showed highly condensed microvasculature composed of tortuous vessels (Fig. 8).

## Discussion

Our unique case series revealed *in vivo* insights about the tumour vascularity using two imaging modalities, preoperative DSC perfusion MRI and intraoperative ultrafast Doppler ultrasound. These findings about the microvascular architecture in both high- and low-grade glioma challenge the current assumption behind the estimation of rCBV regarding the arbitrary distribution of blood vessels in a voxel. Our ultrafast Doppler ultrasound images revealed details of the tumour microvasculature which appears to have a dense well-structured morphology and organised vascular network irrespective of the MRI perfusion state. Additionally, the microvasculature of some of these tumours was seen to a have vascular network seeming to merge into a single vessel.

DSC perfusion MRI has demonstrated a powerful role in neuroradiology practice to clinically and non-invasively assess the histopathological features and vascular characteristics of brain tumours [5, 7, 12, 26]. The rCBV parameter derived from DSC perfusion MRI has been used to differentiate between tumour grades on the basis of the tumour vascularity, where increased malignancy is associated with increased vascularity [12, 27, 28]. Maia et al. [28] evaluated the correlation between rCBV and tumour angiogenesis in glioma using the vascular endothelial growth factor (VEGF) as a marker for angiogenesis. Their results showed that high-grade tumours had a positive VEGF immunoreactivity and significantly correlated to high rCBV values, compared to low-grade tumours with no VEGF expression with low values of rCBV. Furthermore, the study of Huang et al. [29] measured the expression of VEGF and its receptors in different types of brain tumours. Their findings demonstrated that glioblastoma has the highest amount of VEGF protein with high expression of the receptors VEGFR-1 and VEGFR-2 m-RNA that majorly contribute to the high vascular density and angiogenesis of glioblastoma. In accordance with these results, our rCBV maps for glioblastoma showed a high perfusion value of (3.1) compared to high-grade astrocytoma grade 4 with an rCBV value of (1.2). Additionally, Law et al. [30] assessed the sensitivity and specificity of DSC perfusion MRI in grading glioma compared to the conventional MRI, reporting 95% sensitivity and 57.5% specificity with a threshold value of 1.7 for the rCBV ratio. In accordance with these findings, we found that all low-grade glioma in our cohort had an rCBV ratio of < 1.7.

The vascular characteristics of low-grade glioma differ widely between the two histopathological types astrocytoma and oligodendroglioma [13, 31–33]. Oligodendriogliomas are known to have small tortuous vessels while astrocytoma tends to have a microvascular architecture similar to the normal brain vessels. Cha et al. [13] found that low-grade oligodendrogliomas are more vascular compared to low-grade astrocytomas. Their results suggested that both high and low-grade oligodendroglioma displayed higher vascular density and microvascular proliferation in contrast to that seen in low-grade astrocytoma. Our results are in keeping with these findings only in oligodendroglioma, where the ultrafast Doppler ultrasound captured intra-operatively revealed a dense vascular bed as expected, while in the astrocytoma, ultrafast Doppler ultrasound images showed well-structured vasculature and abnormal vessels as opposed to the expected normal brain vessels.

Despite the common use of rCBV in clinical practice to assess tumour grade, this perfusion-derived parameter might still be inaccurate with sensitivity ranging from 55 to 83%, especially in cases of oligodendroglioma [30, 34]. Lev et al. [34] demonstrated in their study that glioma grading using rCBV might be inaccurate when oligodendroglioma is included. Moreover, oligodendroglioma showed high perfusion on rCBV maps not reflective of the histological grading. While this may explain part of the less-than-perfect accuracy of DSC perfusion MRI for tumour grading, we should also consider technical aspects. The assessment of the tumour vascularity using DSC perfusion MRI in the previously published studies relies on the assumption of arbitrary random orientation of vessels with uniform distribution [35, 36]. This is in discrepancy to our observations from the intraoperative ultrafast Doppler ultrasound images, in which we have seen that the tumour vascularity varies in the different histopathological and molecular types of glioma, ranging from a dense transversal compact network to well-structured arraying vessels. The estimation of rCBV in brain tumours may thus be inaccurate, and this may be more pronounced in certain tumour types than others depending on their microvascular architecture. Thus, future work is warranted to optimise the estimation of rCBV for grading and typing glioma by using ultrafast Doppler ultrasound images and to investigate the influence of vessel geometry on the estimation rCBV, while taking into consideration that the assumption of random oriented vessel might still be valid at the microscale level of 100 μm compared to the spatial resolution (200 μm) of the ultrafast Doppler ultrasound.

Recently, several studies have assessed the potential clinical usefulness of intraoperative ultrasound for tumour resection, aiming to achieve maximal safe resection during surgery [37]. In a randomised controlled trial for patients with HGG, Incekara et al. [37] have demonstrated the value of intraoperative Brightness-mode ultrasound where complete contrast-enhanced resection was maximised during ultrasound-guided surgery compared to standard surgery under only MRI neuronavigation. Ultrafast Doppler ultrasound would add as a potential benefit the assessment of tumour remnants not only based on differences in echogeneity (as with Brightness-mode ultrasound) but also vascularisation compared to normal brain tissue. Additionally, the registration between ultrafast Doppler ultrasound images and MR images would be highly beneficial to accurately outline the tumour margin for optimal resection without neurological deficits [38]. In a similar study to ours [14], Imbault et al. [38] evaluated the added value of the ultrafast Doppler ultrasound imaging technique during awake craniotomy, in mapping the functional activity of the brain in patients with glioma. Their ultrafast Doppler ultrasound technique identified different regions of brain activation correlated to the fMRI activation sites. Furthermore, the implementation of ultrafast Doppler ultrasound images combined with other metabolic imaging modalities could be valuable to produce meaningful multi-parametric information about the tumour physiology [39]. Provost et al. [39] demonstrated the pre-clinical oncological application of a hybrid triple imaging modality, incorporating ultrafast Doppler ultrasound with PET-CT in one device to visualise the vasculature and metabolism in rats and mice during tumour growth. The translation of this work to humans—*e.g.*, by using F-18 fluorodeoxyglucose hybrid positron emission tomography/MRI imaging preoperatively—could in turn provide useful insights into the tumour physiology as well as targets for focal and personalised treatment.

Our study has some limitations due to its exploratory nature. First, the lack of histopathological information collected from the tumour samples regarding the neoangiogenetic markers such as VEGF, which would be beneficial for the comparison between the neoangiogenetic markers and the different pattern of tumour vasculature observed intraoperatively by the ultrasound images. Second, the lack of direct registration of the ultrafast Doppler ultrasound images to MRI in order to perform exact spatial cross-comparison between the two imaging modalities. In future work, we aim to use neuronavigation software to spatially register the intraoperative ultrafast Doppler ultrasound to the preoperative MRI [40]. A third limitation is the small sample size, but at the same time, this is a case series of unprecedented data and includes the full spectrum of different molecular and histopathological types and grades of glioma.

In conclusion, our ultrafast Doppler ultrasound images revealed details about the tumour vascular bed showing rich vascularisation also in tumours with low perfusion on MRI. These findings, sowing some doubts about the assumptions regarding the vessel distribution and orientation for rCBV estimation, warrant further investigation of DSC MRI postprocessing, in particular for typing and grading adult-type diffuse glioma.

## Data Availability

All data generated or analysed during this study are available from the corresponding author on reasonable request.

## Abbreviations

3D: Three-dimensional
CBV: Cerebral blood volume
DSC: Dynamic susceptibility contrast
fUS: Functional ultrasound
GRE: Gradient-echo
HEPI: Hybrid echo-planar imaging
HGG: High-grade glioma
IDH: Isocitrate dehydrogenase
LGG: Low-grade glioma
MRI: Magnetic resonance imaging
NAWM: Normal appearing white matter
rCBV: Relative cerebral blood volume
VEGF: Vascular endothelial growth factor
VEGFR: VEGF receptor

## References

[1] Louis DN, Perry A, Wesseling P (2021). The 2021 WHO Classification of Tumors of the Central Nervous System: a summary. Neuro Oncol.

[2] Aronen HJ, Perkiö J (2002). Dynamic susceptibility contrast MRI of gliomas. Neuroimaging Clin N Am.

[3] Law M, Yang S, Babb JS (2004). Comparison of cerebral blood volume and vascular permeability from dynamic susceptibility contrast-enhanced perfusion MR imaging with glioma grade. AJNR Am J Neuroradiol.

[4] Covarrubias DJ, Rosen BR, Lev MH (2004). Dynamic magnetic resonance perfusion imaging of brain tumors. Oncologist.

[5] Aronen HJ, Gazit IE, Louis DN (1994). Cerebral blood volume maps of gliomas: comparison with tumor grade and histologic findings. Radiology.

[6] Gillard JH, Waldman AD, Barker PB (2009). Clinical MR neuroimaging.

[7] Sugahara T, Korogi Y, Kochi M (1998). Correlation of MR imaging-determined cerebral blood volume maps with histologic and angiographic determination of vascularity of gliomas. AJR Am J Roentgenol.

[8] Knopp EA, Cha S, Johnson G (1999). Glial neoplasms: dynamic contrast-enhanced T2*-weighted MR imaging. Radiology.

[9] Abrigo JM, Fountain DM, Provenzale JM et al (2018) Magnetic resonance perfusion for differentiating low-grade from high-grade gliomas at first presentation. Cochrane Database Syst Rev 2018. 10.1002/14651858.CD011551.pub2 10.1002/14651858.CD011551.pub2 PMC6491341 29357120

[10] van Santwijk L, Kouwenberg V, Meijer F (2022). A systematic review and meta-analysis on the differentiation of glioma grade and mutational status by use of perfusion-based magnetic resonance imaging. Insights Imaging.

[11] Provenzale JM, Wang GR, Brenner T (2002). Comparison of permeability in high-grade and low-grade brain tumors using dynamic susceptibility contrast MR imaging. AJR Am J Roentgenol.

[12] Hakyemez B, Erdogan C, Ercan I (2005). High-grade and low-grade gliomas: differentiation by using perfusion MR imaging. Clin Radiol.

[13] Cha S, Tihan T, Crawford F (2005). Differentiation of low-grade oligodendrogliomas from low-grade astrocytomas by using quantitative blood-volume measurements derived from dynamic susceptibility contrast-enhanced MR imaging. AJNR Am J Neuroradiol.

[14] Soloukey S, Vincent AJPE, Satoer DD et al (2020) Functional ultrasound (fUS) during awake brain surgery: the clinical potential of intra-operative functional and vascular brain mapping. Front Neurosci 13. 10.3389/fnins.2019.01384 10.3389/fnins.2019.01384 PMC6962116 31998060

[15] Arzanforoosh F, van der Voort SR, Incekara F (2023). Microvasculature features derived from hybrid EPI MRI in non-enhancing adult-type diffuse glioma subtypes. Cancers (Basel).

[16] Klein S, Staring M, Murphy K (2010). elastix: a toolbox for intensity-based medical image registration. IEEE Trans Med Imaging.

[17] Isensee F, Schell M, Pflueger I (2019). Automated brain extraction of multisequence MRI using artificial neural networks. Hum Brain Mapp.

[18] Isensee F, Jaeger PF, Kohl SAA (2021). nnU-Net: a self-configuring method for deep learning-based biomedical image segmentation. Nat Methods.

[19] Kickingereder P, Isensee F, Tursunova I (2019). Automated quantitative tumour response assessment of MRI in neuro-oncology with artificial neural networks: a multicentre, retrospective study. Lancet Oncol.

[20] Luu HM, Park SH (2022) Extending nn-UNet for Brain Tumor Segmentation. In: Crimi A, Bakas S (eds) Brainlesion: Glioma, Multiple Sclerosis, Stroke and Traumatic Brain Injuries. BrainLes 2021. Lecture Notes in Computer Science, vol 12963. Springer, Cham. 10.1007/978-3-031-09002-8_16

[21] McKinley R, Rebsamen M, Dätwyler K, Meier R, Radojewski P, Wiest R (2021) Uncertainty-Driven Refinement of Tumor-Core Segmentation Using 3D-to-2D Networks with Label Uncertainty. In: Crimi A, Bakas S (eds) Brainlesion: Glioma, Multiple Sclerosis, Stroke and Traumatic Brain Injuries. BrainLes 2020. Lecture Notes in Computer Science, vol 12658. Springer, Cham. 10.1007/978-3-030-72084-1_36

[22] Arzanforoosh F, Croal PL, van Garderen KA et al (2021) Effect of applying leakage correction on rCBV measurement derived from DSC-MRI in enhancing and nonenhancing glioma. Front Oncol 11. 10.3389/fonc.2021.648528 10.3389/fonc.2021.648528 PMC8044812 33869047

[23] Zhang Y, Brady M, Smith S (2001). Segmentation of brain MR images through a hidden Markov random field model and the expectation-maximization algorithm. IEEE Trans Med Imaging.

[24] Jenkinson M, Smith S (2001). A global optimisation method for robust affine registration of brain images. Med Image Anal.

[25] Jenkinson M (2002). Improved optimization for the robust and accurate linear registration and motion correction of brain images. Neuroimage.

[26] Romano A, Rossi Espagnet MC, Calabria LF (2012). Clinical applications of dynamic susceptibility contrast perfusion-weighted MR imaging in brain tumours. Radiol Med.

[27] Zhou J, Li N, Yang G, Zhu Y (2011). Vascular patterns of brain tumors. Int J Surg Pathol.

[28] Maia ACM, Malheiros SMF, da Rocha AJ (2005). MR cerebral blood volume maps correlated with vascular endothelial growth factor expression and tumor grade in nonenhancing gliomas. AJNR Am J Neuroradiol.

[29] Huang H, Held-Feindt J, Buhl R (2005). Expression of VEGF and its receptors in different brain tumors. Neurol Res.

[30] Law M, Yang S, Wang H (2003). Glioma grading: sensitivity, specificity, and predictive values of perfusion MR imaging and proton MR spectroscopic imaging compared with conventional MR imaging. AJNR Am J Neuroradiol.

[31] Schiffer D, Bosone I, Dutto A (1999). The prognostic role of vessel productive changes and vessel density in oligodendroglioma. J Neurooncol.

[32] Schiffer D, Dutto A, Cavalla P (1997). Prognostic Factors in Oligodendroglioma. Can J Neurol Sci.

[33] Guo H, Kang H, Tong H (2019). Microvascular characteristics of lower-grade diffuse gliomas: investigating vessel size imaging for differentiating grades and subtypes. Eur Radiol.

[34] Lev MH, Ozsunar Y, Henson JW (2004). Glial tumor grading and outcome prediction using dynamic spin-echo MR susceptibility mapping compared with conventional contrast-enhanced MR: confounding effect of elevated rCBV of oligodendrogliomas [corrected]. AJNR Am J Neuroradiol.

[35] Quarles CC, Gochberg DF, Gore JC, Yankeelov TE (2009). A theoretical framework to model DSC-MRI data acquired in the presence of contrast agent extravasation. Phys Med Biol.

[36] Pathak AP, Ward BD, Schmainda KM (2008). A novel technique for modeling susceptibility-based contrast mechanisms for arbitrary microvascular geometries: The finite perturber method. Neuroimage.

[37] Incekara F, Smits M, Dirven L et al (2021) Intraoperative B-mode ultrasound guided surgery and the extent of glioblastoma resection: a randomized controlled trial. Front Oncol 11. 10.3389/fonc.2021.649797 10.3389/fonc.2021.649797 PMC8170308 34094939

[38] Imbault M, Chauvet D, Gennisson J-L (2017). Intraoperative functional ultrasound imaging of human brain activity. Sci Rep.

[39] Provost J, Garofalakis A, Sourdon J (2018). Simultaneous positron emission tomography and ultrafast ultrasound for hybrid molecular, anatomical and functional imaging. Nat Biomed Eng.

[40] Soloukey S, Verhoef L, Jan van Doormaal P et al (2022) High-resolution micro-Doppler imaging during neurosurgical resection of an arteriovenous malformation: illustrative case. J Neurosurg Case Lessons 4. 10.3171/CASE22177 10.3171/CASE22177 PMC9644416 36345205

